# Rectal stromal tumor with an exceptional liver and bone metastatic locations

**DOI:** 10.11604/pamj.2019.32.133.17985

**Published:** 2019-03-20

**Authors:** Dougaz Mohamed Wejih, Gouta Esma Leila, Chaouch Mohamed Ali, Nouira Ramzi, Bouasker Ibtissem, Dziri Chadli

**Affiliations:** 1 Surgery B Department, Charles Nicolle Hospital, Tunis, Tunisia

**Keywords:** Stromal tumor, metastases, gastrointestinal neoplasm

## Abstract

Gastrointestinal stromal tumours (GIST) are rare mesenchymal tumours which represent 1% to 3% of gastrointestinal neoplasm. Rectal location of GIST is extremely rare reaching 5% of GIST and only 0.1% of rectal tumours. They usually metastases to the liver (65%) and exceptionally to the bone (3%). We reported a case of rectal stromal tumour with an exceptional metastasis located in the rib. A 40-year-old man who presented with pelvic pain, associated with rectal syndrome, rectal bleeding and subocclusive episodes. Physical examination objectified a tough, budding rectal mass, with a smooth wall, localized 3cm above of anal margin. A Thoraco-abdominal computed tomography showed a large heterogeneous tissue mass, taking the whole pelvis, coming from the right-side wall of the rectum of 17.3 x 14cm. It was associated with liver and bone secondary locations. Biopsies confirmed the secondary locations of an intermediate risk GIST. Immunohistochemical study showed an overexpression of c-kit protein (CD117) and Dog1. Imatinib was prescribed to reduce the tumour size. Stromal metastatic rectal tumours in bone level are extremely rare conditions. The diagnosis is confirmed by histological examination with immune histochemical analysis. The prognosis remains poor in metastatic forms but it has been improved since the introduction of Imatinib.

## Introduction

Gastrointestinal stromal tumours (GIST) are rare mesenchymal tumours. They represent 1% to 3% of gastrointestinal neoplasm [1]. GIST are located preferentially at the stomach (55%) [2], followed by small intestine (31%) [2]. The rectal location of GIST is extremely rare. It represents 5% of GIST and only 0.1% of rectal tumours [3]. The most common metastatic sites are the liver (65%). Bone metastases are extremely rare and the treatment is based on targeted drug therapy (3%) [4]. We reported a case of a localized stromal tumour in the rectum, with liver and bone metastases.

## Patient and observation

**Figure 1:**
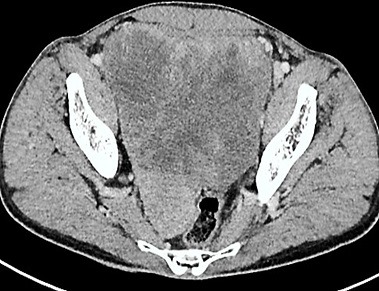
Abdominal CT scan showing a large pelvic mass

**Figure 2:**
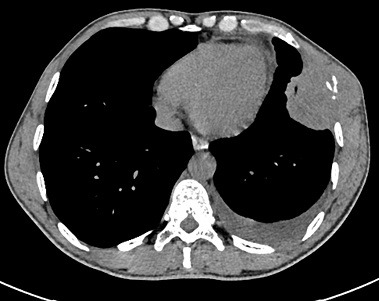
Chest CT scan showing a heterogeneous mass localized in the 6^th^ left rib

**Figure 3:**
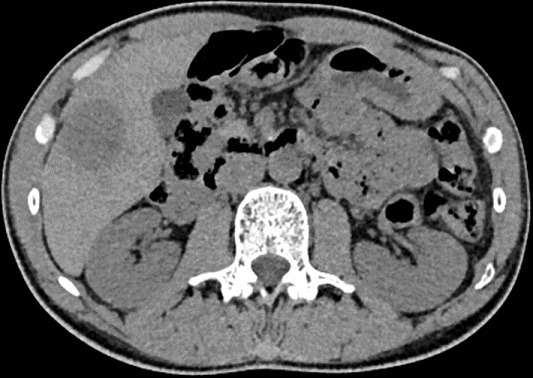
Abdominal CT scan showing a round hypodense lesion localized in the liver segment VI

**Figure 4:**
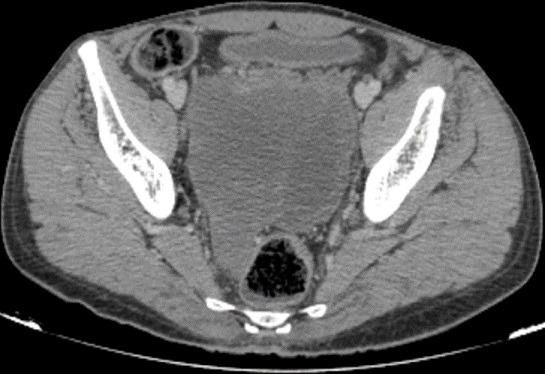
Abdominal CT scan showing rectal’s tumour size regression

**Figure 5:**
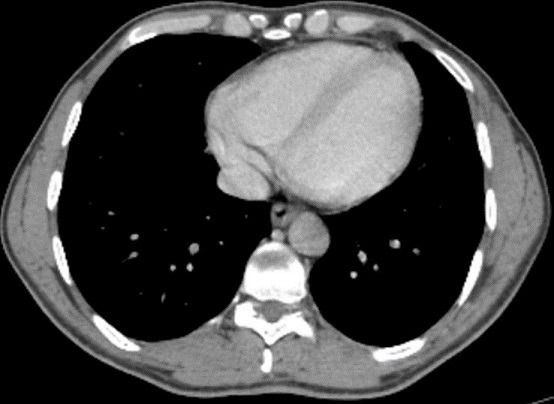
Chest CT scan showing disappearance of the rib metastasis

**Figure 6:**
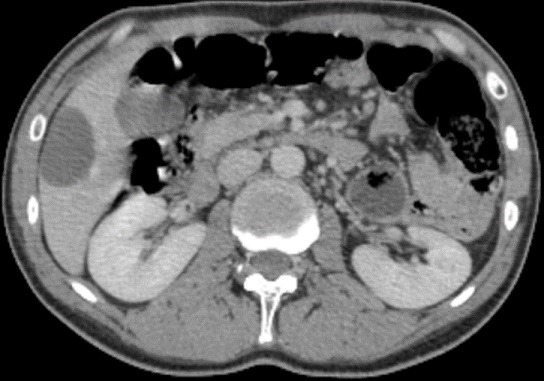
Abdominal CT scan showing a partial response of the liver metastasis to treatment

A 40-year-old man, with no medical history, presented pelvic pain for three months, associated with a rectal syndrome, rectal bleeding and subocclusive episodes which were resolved spontaneously. There was no concept of impaired general condition. Physical examination objectified a tough, budding rectal mass, with a smooth wall, localized 3cm above of anal margin. The chest examination showed a mass of 5cm which was tough, painful, situated next to the 6th left rib. A colonoscopy showed extrinsic compression of the rectum without evidence of a rectal tumour mass. A thoraco-abdominal computed tomography showed a large tissue mass taking the whole pelvis, with heterogeneous enhancement and presence of multiple necroses ranging of 17.3 x 14cm. It seemed to come from the right-side wall of the rectum (Figure 1). It is associated with liver and bone secondary locations (Figure 2, Figure 3). Biopsies of liver and bone metastasis confirmed the secondary locations of an intermediate risk GIST. Immunohistochemical study showed an overexpression of c-kit protein (CD117) and Dog1. Imatinib was prescribed in order to reduce the tumour size before proposing surgical resection. The abdominal CT scan, performed 3 months after starting treatment, showed a good response with regression of the rectal tumour and the liver metastasis and disappearance of the rib metastasis (Figure 4, Figure 5, Figure 6).

## Discussion

Our case showed an uncommon metastasis location of GIST in the liver and bones. GISTs are tumours growing from connective tissue support. The most common metastatic sites are the liver (65%) and the peritoneal surface (50%) [4]. Cases of bone metastasis are rare, with 12 reported cases in the literature [4]. The most frequent sites of bone metastases reported in many case series were spine and pelvis. Our patient presented bone metastasis localized in the ribs. Bone metastasis can cause pain or lead to a pathological fracture, which may impair mobility and social functioning. Therefore, we should be aware that bone metastases can occur in patients with metastatic GIST and treat them on time before serious complications occur [5]. In our case, the patient didn't complain about bone pain. Abdominal CT scan is the best imaging for diagnosis, staging and post-operative care. It shows a heterogeneous and well-limited mass, with mostly exoluminal development, with occasional necrotic and haemorrhagic alterations [6]. The cystic lesion may be thick-walled. There are no specific characteristics to GISTs in CT imaging [6]. Pathological examination with immune histo-chemical analysis confirms the diagnosis by immune marking CD117 (c-kit) and CD34. Surgical resection is the only curative treatment for localized tumours [7-9]. Imatinib is used as part of an adjuvant therapy or in the metastatic forms. The prognosis has improved since the introduction of imatinib, particularly as adjunctive therapy, but it is poor in metastatic forms [10]. The 5-year disease-free survival rate for metastatic GIST after resection is about 43.9% [11]. In addition to Imatinib, radiotherapy and Bisphosphonates can also be used in patients with bone metastases as a palliative therapy. However, the optimal treatment for bone metastases originating from GIST is yet to be elucidated.

## Conclusion

Stromal metastatic rectal tumours in bone level are extremely rare conditions. The diagnosis is confirmed by histological examination with immune- histochemical analysis. The prognosis remains poor in metastatic forms but it has been improved since the introduction of Imatinib.

## Competing interests

The authors declare no competing interests.
